# Chloride accumulation in aboveground biomass of three macrophytes (*Phragmites australis*, *Juncus maritimus,* and *Typha latifolia*) depending on their growth stages and salinity exposure: application for Cl^−^ removal and phytodesalinization

**DOI:** 10.1007/s11356-021-17591-3

**Published:** 2022-01-20

**Authors:** Emmanuel Delattre, Isabelle Techer, Benjamin Reneaud, Patrick Verdoux, Isabelle Laffont-Schwob, Philippe Prohin

**Affiliations:** 1 UPR CHROME, Université de Nîmes, rue du Dr. Georges Salan, 30021 Nimes, France; 2 Aix Marseille Univ, IRD, LPED, Marseille, France; 3 Nymphéa Distribution, 30740 Le Cailar, France

**Keywords:** Salinity tolerance, Chloride bioaccumulation, Chloride removal, Phytodesalinization, Phytoremediation, Growth Stage, Common reed, Sea rush, Cattail

## Abstract

**Abstract:**

Anthropogenic activities can be the source of saline solid wastes that need to be treated to reduce their salt load to meet the purposes of reuse, valorization or storage. In this context, chloride remediation can be achieved using high-salt accumulating plants. However, there is very limited information on the comparative potential of different species in the same environment, and only scarce data concerning their efficiency as a function of growth stage. In order to rationalize these selection criteria, three macrophytes i.e., common reed (*Phragmites australis)*, sea rush (*Juncus maritimus*), and cattail (*Typha latifolia*), were cultivated at two growth stages (6-months old and 1-year old) for 65 days in Cl^−^ spiked substrates (from 0 up to 24 ‰ NaCl). The plants’ survival and potential capacity for removal of Cl^−^ from substrates and accumulation in shoots were investigated. For the three studied species, mature and juvenile plants display a high tolerance to salinity. However, mature specimens with higher shoot biomass and Cl^−^ contents are capable of greater chloride removal than juvenile plants. The sole exception is *P.* *australis* which displays just the same phytoremediation potential for both mature and juvenile specimens. Moreover, *P. australis* has the lowest potential when compared with other species, being 1.5 and 3 times lower than for *J. maritimus* and *T. latifolia*. When considering the plant growth and the shoot biomass production, chloride removal rates from the substrate point that mature *J. maritimus* should preferentially be used to design an operational chloride remediation system. The results highlight the relevance of considering the growth stage of plants used for Cl^−^ removal.

**Highlights:**

1) Mature and juvenile specimens of *J. maritimus*, *P. australi*s, and *T. latifolia* have high salinity tolerance in solid media spiked up to 24 ‰ NaCl.

2) Mature plants have generally better Cl^−^ removal and phytoremediation performances than juvenile specimens.

3) *J. maritimus* is the most effective species for chloride phytoremediation with high survival and high Cl^−^ sequestration in shoots.

4) *T. latifolia* has high Cl^−^ removal in shoots and good remediation capacities but also shows sign of stress.

5) *P. australis* shows low Cl^−^ sequestration and is a poor candidate for chloride remediation from substrate.

**Supplementary Information:**

The online version contains supplementary material available at 10.1007/s11356-021-17591-3.

## Introduction

Soil salinization represents a major environmental issue of our time. Being an extensive phenomenom which involves the accumulation of high concentrations of saline and sodic ions, soil salinization is expected to affect more than 830 millions hectares of the continents (Daliakopoulos et al. 2016; Ivushkin et al. 2019). While salinization is known to be primarily caused by natural processes (primary salinization) that are accentuated by global climate changes, it can also be induced or enhanced by human activities, mainly irrigation (secondary salinization; Daliakopoulos et al. 2016). In a same way, many industrial processes (e.g., oil, paper and pulp industries, cement manufacturing, aquaculture, textile treatments, and application of road salts) can promote the salinity of natural ecosystems through the production of saline effluents (for review, see Litalien and Zeeb 2020). In addition, many anthropogenic activities generate saline solid wastes, including sediment and mud. For instance, harbour dredging is pointed out as a maintenance activity producing large amounts of saline sediments whose management is clearly challenging (Wang et al. 2018). This global context of salinization and the environmental issues raised have led to setting up of new regulations which promote the remediation of saline effluents, saline wastes and marine sediments, with the aim of protecting the environment. Among the ions involved in salinization, chloride is specifically targeted by these regulations, being considered harmful to ecosystems. For instance, chloride can make up more than 1% of the chemical composition of dredged sediments (Lim et al. 2020). The management of chloride-rich sediments and wastes is challenging as it is inconsistent with many industrial technologies classically used to treat and valorize these materials. High chloride contents are incompatible with most physical, thermal and chemical industrial remediation processes (leading to the degradation of the industrial facilities). Waste storage solutions are also drastically limited by the chloride concentrations (threshold on leachates set at 800 mg.kg^−1^ for inert wastes, 15,000 mg.kg^−1^ for non-inert and non-hazardous wastes and 25,000 mg.kg^−1^ for hazardous wastes according to the French regulations). It would be of great interest to use high-salt accumulating plants to phytoremediate chloride-rich soils, effluents, and wastes since this passive treatment process has a low ecological and economic imprint (Jesus et al. 2015).

Salt phytoremediation can simply be defined as “the cultivation of salt accumulating or salt-tolerant plants for the reduction of […] salinity and/or sodicity” (Qadir and Oster 2002). The phytoremediation of saline environments has thus a special character since it requires the use of plant species able to survive and adapt to saline ions in excess, and which can control saline ion concentrations through various mechanisms. Only halophytes species (i.e., about 1% of plant species, Flowers 2008) can be considered for this remediation purposes. Halophytes are salt tolerant species due to various adaptation mechanisms such as the exclusion of salt ions in excess at the root level, the uptake and sequestration of these ions in cells of the aerial organs (accumulator halophytes), or the ion excretion at leaf surfaces (recretohalophytes) (Yensen and Biel 2006). Most of the halophyte species studied for their ability to remediate salt-affected soils and effluents are accumulators (Brown et al. 1999; Doni et al. 2015; Jesus et al. 2014; Khandare and Govindwar 2015; Manousaki and Kalogerakis 2011; Masciandaro et al. 2014; Padmavathiamma et al. 2014; Pouladi et al. 2016; Qadir et al. 2007; Rabhi et al. 2009). These studies focus on the capacity of accumulators to sequester saline ions in the aboveground biomass. In recent years, some studies have dealt with the potential of recretohalophytes to remediate saline environments (Litalien and Zeeb 2020; McSorley et al. 2016a). Investigations are needed to validate the applicability of this last phenotype for depollution.

Hence, the most studied mechanism involved in the remediation of saline environments is the vacuolar sequestration of excess ions in the cells of the aerial parts of halophytes. Sequestration of excess cations, mainly Na^+^, is generally studied with respect to their toxicity for plants. In contrast, sequestration of Cl^−^ anions is less well documented. Chloride is a micronutrient useful for plants, e.g. in photosynthesis, but can be phytotoxic at high levels (White and Broadley 2001). Some halophyte species are known for their capacity to accumulate chloride (Devi et al. 2016, 2008; Krishnapillai and Ranjan 2005; Rabhi et al. 2009, 2008; Hasanuzzaman et al. 2014). In this context, the most studied species are *Atriplex* spp*., Avicennia* spp., *Phragmites* spp., *Salicorna* spp., *Sesuvium* spp., *Suaeda* spp., *Tetragonia* spp., and *Typha* spp. For example, Cl^−^ ion concentrations in shoots can reach 70 mg.g^−1^ DW (dry weight) in *Suaeda maritima* (L.) Dumort., 20 mg.g^−1^ DW in *Aeluropus littoralis* (Gouan) Parl. (Hasanuzzaman et al., 2014), 24 mg.g^−1^ DW in *Phragmites australis* (Cav.) Trin. ex Steud. (McSorley et al. 2016b), 20 mg.g^−1^ in *Juncus maritimus* Lam. (Al Hassan et al. 2016) and range between 24 mg.g^−1^ and up to 63 mg.g^−1^ in *Typha latifolia* L. (Morteau et al. 2009; Rozema et al. 2014). By combining their high potential of Cl^−^ accumulation and relatively high biomass, some halophytes can be used in phytoremediation to treat soils and effluents (Fountoulakis et al. 2017; Gorai et al. 2010; Guesdon et al. 2016; Jesus et al. 2015, 2017; McSorley et al. 2016b; Morteau et al. 2009; Calheiros et al. 2009, 2008; Rozema et al. 2014). However, little is known about how the age of a plant affects its aclimation to harmful saline media and its capacity to take up and store ions, and specifically chloride. In this framework, most of the studies deal only with the effect of salinity on seed germination (Boscaiu et al. 2011; Davy et al. 2006; Wu et al. 2016; Yu et al. 2012). To our knowledge, few authors gives any information on salt tolerance and Cl^−^ ion sequestration as a function of plant growth stage (Chartzoulakis and Klapaki 2000; Zeng et al. 2002). Similarly, phytoremediation studies rarely report the growth stage at which plants are included in experiments. However, this criteria should be determinant in the choice of a phytoremediation model. For instance, Lissner and Schierup (1997) observed a higher mortality of juvenile plants of *P. australis* (10-weeks old) compared to larger rhizome-grown plants when cultivated in saline solutions. The challenge here is to define at which stage of growth specimens should be implanted in a system to be treated.

This study was performed in order to collect data on the tolerance of macrophyte species to Cl^−^-spiked substrates as well as on chloride accumulation and removal depending on their growth stage. The aim is to provide sufficient data to discuss the chloride removal potential of a given species as a function of growth stage at the time of its introduction into a saline matrix. Three macrophytes were selected as being tolerant to a large range of salinity and also because they are chloride accumulators used in soil and effluent remediation experiments: *P. australis*, *J. maritimus*, and *T. latifolia*. *P. australis* is a cosmopolitan glycophyte commonly used in wastewater treatment. Although it is not a true halophyte, it can tolerate environments with salinities up to 23 ‰ NaCl for haplotype M and 6 ‰ for others haplotypes (Vasquez et al. 2005). As mentioned above, it does not accumulate high contents of Cl^−^ in its shoots, but compensates with a high aboveground biomass (1 to 5.5 kg.m^−2^) (McSorley 2016a; Moore et al. 2012; Rozema et al. 2016). *J. maritimus* is less well documented, but is known to be tolerant to high salinities up to 30 ‰ NaCl (Boscaiu et al. 2011, 2007). In contrast, *T. latifolia* is mostly encountered in habitats with moderate salinity, between 12 ‰ and 24 ‰ NaCl but with better salt removal capacity (Rozema et al. 2016, 2014).

While these three species are good candidates to remediate saline media, effluents or soils (Fountoulakis et al. 2017; Guesdon et al. 2016; Jesus et al. 2015, 2017; McSorley et al. 2016b; Morteau et al. 2009; Calheiros et al. 2009, 2008; Rozema et al. 2014; Prabakaran et al. 2019) it is difficult to determine which one is best suited for a given environment due to a lack of uniformity in the literature data where each species has often been studied separately under different experimental conditions. In this study, *P. australis*, *J. maritimus* and *T. latifolia* plants were cultivated at two growth stages in five distinct chloride concentrations in order to compare their ability to remediate saline environment and study the importance of the growing stage in this context.

## Materials and methods

### Plants

Seeds of *Phragmites australis* (Cav.) Trin. ex Steud. (Poaceae), *Typha latifolia* L. (Typhaceae) and *Juncus maritimus* Lam. (Juncaceae) were sown and the seedlings cultivated by an aquatic plant production company, Nymphea Distribution (France, Le Cailar, 30,740). Plants were harvested at two growth stages, 6 months and 1 year, as defined by their cultivation duration in the company greenhouses. These two sets of plants were called “juvenile” (j) and “mature” (m) respectively. The plants were collected along with their own substrate, composed of clays, black and white peat, as well as organic fertilizers such as dried poultry blood and guano (exact composition known but kept confidential for industrial reasons). The cultural practices of Nymphea Distribution are described in Delattre et al. (2020).

### Experimental conditions

To compare between species and growth stages, the three studied macrophytes were introduced separately into plastic food containers, called microcosms. Due to the plant clod size, the container dimensions were of 12 x 16 x 10 cm for juvenile plants, and 20 x 20 x 10 cm for mature plants. For each species and each growth stage, four microcosms were observed being characterized by a given chloride enrichment. A given volume of 20 g.L^−1^ NaCl solution was introduced to yield substrate chloride concentrations of 1,875 mg.kg^−1^ (microcosm C1, 3‰ NaCl), 3,750 mg.kg^−1^ (microcosm C2, 6‰ NaCl), 7,500 mg.kg^−1^ (microcosm C3, 12‰ NaCl) and 15,000 mg.kg^−1^ (microcosm C4, 24‰ NaCl). These chloride contents from 1,875 up to 15,000 mg.kg^−1^, were selected as being close to critical threshold concentrations expected for waste storage according to French regulations. Microcosms were set up in triplicate for juvenile plants (12 plants/microcosm) and in duplicate for mature plants (9 plants/microcosm). For each set of juvenile and mature plants, a control microcosm was studied that was not enriched in NaCl (C0).

**Fig. 1:**
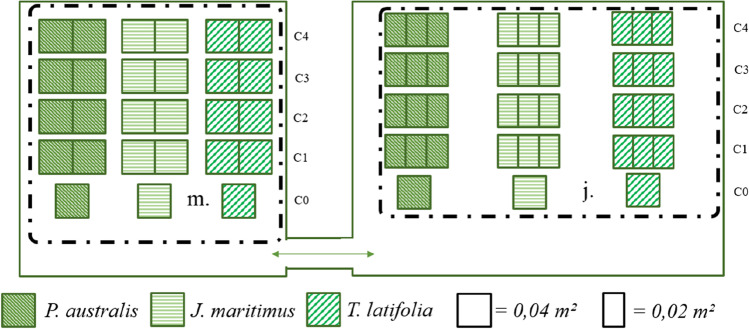
Scheme of the cultivation rooms and disposition of the experimental microcosms. The arrow shows the air circulation. Microcosms are considered as a function of the substrate chloride contents in mg.kg^−1^ Cl^−^ and ‰ NaCl (C0 = 0; C1 = 1,875 mg.kg^−1^, 3‰; C2 = 3,750 mg.kg^−1^, 6‰; C3 = 7,500 mg.kg^−1^, 12‰; C4 = 15,000 mg.kg^−1^, 24‰), the plants species (*P. australis, J. maritimus* and *T. latifolia*) and their growth ages (m. = mature plants, j. = juvenile plants)

The cultivation was maintained under controlled environmental conditions in two cultivation rooms (Growbox©) for 80 days (between 27th April and 16th July, 2018) (Fig. 1). The dimension of these rooms did not allow the study of triplicates for mature plants. The two rooms communicated through a vent and were equipped with fans to homogenize the atmosphere and mimic wind effects on shoots (plant anemomorphosis). The photoperiod was set at 12 h with 400 W lamp HPI-T Plus (400 W/645 E40, PHILIPS), the hygrometry and temperature was monitored with a TESTO 175H1 probe (T: -20 to + 55 ± 0.4 °C, %RH: 0 to 100 ± 2%). The mean day temperature and hygrometry (between 6 a.m. and 6 p.m.) were respectively of 24.00 ± 2.20 °C (max = 29.2, min = 14.1) and 57.18 ± 8.03% (max = 79.9, min = 31.0) while the mean night temperature and hygrometry (between 6 p.m. and 6 a.m.) were respectively of 21.39 ± 2.25 °C (max = 29.2, min = 14.0) and 63.90 ± 9.98% (max = 80.6, min = 32.1).

After NaCl enrichment, the plants were irrigated every two days to obtain a constant volume of 800 mL of water in each microcosm. The water used for irrigation was that initially introduced into the production system by the Nymphea Distribution company. This water was pumped from a local aquifer (groundwater from the Vistrenque aquifer) each week and stored in a 250 L container in HDPE for the purpose of the experiments. The chloride concentration of this irrigation water was determined over the course of the experiment and was of 62.7 ± 0.5 mg.L^−1^ (2σ, *n* = 9). This result is in agreement with the value published by Sassine et al. (2015) for the Vistrenque groundwater. The volume of water added to the microcosms throughout the experiment led to a chloride “natural” flux input of approximately 1.3 ± 0.4 g (1σ) and 1.4 ± 0.5 g (1σ) for the j and m microscosms, respectively.

### Plant sampling

Following the NaCl addition (t0), a first step of 2 weeks was adopted for plant aclimation. During this step, cultivation was maintained but no specimens were sampled. An initial shoot sampling was carried out before the adaptation period of 15 days (t0) and also afterwards (t0bis) to determine the initial Cl^−^ shoot contents. Plants were then collected at day 3, 9, 15, 25, 36, 45, and 65 after aclimation periode (thereafter denoted as t3, t9, t15, t25, t36, t45, and t65). The number of individuals sampled at each date was chosen to ensure sufficient biomass to allow measurements in triplicate. For the first three sampling dates, 2 plants were collected in each triplicate juvenile microcosms and 1 plant in the duplicate mature microcosms. For the following sampling dates, 1 plant was collected in each triplicate and duplicate microcosm for the juvenile and mature plants, respectively. At day 65, the remaining plants were collected (9 specimens of juvenile plants; 2 specimens of mature plants). Only the aboveground biomass was sampled to study Cl^−^ removal by phytoextraction simulating an industrial process that would take into account the growth of plants on the substrate to be treated and removal of the aerial parts, thus allowing new growth from resprouting. In such an industrial process, roots are not harvested to avoid replantation that would increase the operational expenditure.

### Plant sample treatment and analysis

The shoots were cut and then dried at 75 °C for at least 48 h. Fresh and dry mass was determined before and after drying to determine plant water contents. The biomass data are expressed in dry weight (DW) in the results section. Dried samples were then crushed with a ball mill (SPEX) and the powder sieved at 200 µm (Pavlik 2000). The fine fraction was used to determine the chloride concentration in shoots by cold water extraction according to McSorley et al. (2016b). The chloride concentration of shoot extracted solutions was determined by silver titration. A solution of 0.01 M AgNO_3_ was prepared with a coloured indicator of 0.3 M potassium chromate. A volume of extracted solution between 0.5 and 2 mL was introduced into a beaker and titrated to determine the chloride concentration with an electronic bottletop titrator. Chloride concentrations are expressed in mg.g^−1^ DW.

### Evaluation of survival rates

The resistance and survival of species in each saline media were evaluated by counting the number of living and dead plants every two days during the experiment. Criteria for evaluating dead plants were senescence, lack of resprouting, stiffness and water content at touch (soaked when dead). The survival rate cannot be determined with classical methods because plant sampling in the microcosms over the duration of the experiment led to the occurrence of censored values. As a result, the survival rate was assessed with the Kaplan–Meier estimator (Ŝ(t)) (Kaplan and Meier 1958) (Eq. 1).

1 $$\widehat S\left(t\right)={\textstyle\prod_{i:t_i\leq t}}(1-d_i/n_i)$$

With:

t_i_, the time of observation,

d_i_, the number of dead plants at the time t_i_,

n_i_, the number of plants known to survive (not included dead or censored).

### *Total shoot Cl*^*−*^* contents*

To determine the total amount of chloride taken up by the plants in a given microscosm at the end of the experiment (in the 9 mature plants and 12 juvenile plants of the studied microcosms) (in mg of Cl^−^), the chloride contents (mg.g-1 DW) added at each time of sampling as well as the mass of aboveground collected shoots (g DW) were measured. The total chloride uptake is the sum of Cl^−^ accumulated in each sample as expressed by the following equation:

2 $${AC}_T=\sum\nolimits_{ti=0}^{65}({\left[{Cl}^-\right]}_{ti}-{\left[{Cl}^-\right]}_{t-15})\times{mb}_{ti}$$

With:

${AC}_{T}$, the total chloride amount accumulated in shoots of a given microscosm at the end of the experiment (in mg),

mb_ti_, the shoot biomass of the samples at time ‘i’ (g DW),

${\left[{Cl}^{-}\right]}_{ti}$, the shoot Cl^−^ concentration of the samples at time ‘i’ (mg.g^−1^ DW),

${\left[{Cl}^{-}\right]}_{t-15}$, the initial shoot Cl^−^ concentration measured at time ‘t0bis’ (mg.g^−1^ DW).

ti, time of sampling.

### Chloride removal from the substrate

The chloride remediation of the substrate was determined by normalizing the total Cl^−^ amount accumulated in the shoots to the total Cl^−^ amount in the microcosm substrate at the beginning of the experiment. Taking into account Cl^−^ inputs from the irrigation water, a removal ratio (expressed in %) can be defined using Eq. 3 modified from McSorley et al. (2016b). As previously mentioned, this approach only accounts for chloride removal leading to accumulation in the shoots. Roots and rhizomes are not studied here as their harvest would not be required in an industrial production system.

3 $$R=\frac{{AC}_{T}}{{(\left[{Cl}^{-}\right]}_{m}\times {m}_{m})+{(\left[{Cl}^{-}\right]}_{w}\times {V}_{w})}$$

With:

R, the shoot chloride removal rate in a given microcosm (in %).

${AC}_{T}$, the total shoot Cl^−^ accumulation (in mg per microcosm),

[Cl^−^]_m_ the initial Cl^−^ concentration in substrate of the microcosm (mg.kg^−1^),

m_m_ the mass of the substrate in the microcosm (kg),

[Cl^−^]_w_ the Cl^−^ concentration of the irrigation water (mg.L^−1^),

V_w_ the volume of irrigation water added to the microcosm during the course of the experiment (L).

The theoretical chloride removal (R_t_) can also be calculated assuming a scenario with no plant sampling over the experiment duration. This takes into account *n* plants in each microcosm (*n* = 9 or 12), by considering the biomass and shoot Cl^−^ content measured at the end of the experiment (Eq. 4). In this scenario, the increase of biomass is assumed to be negligible, so the biomass measured at time t_i_ can be taken as constant throughout the experiment; in fact, no significant growth of the plants was observed over the experiment duration (suppl. Information—Table 1).

4 $$R_t=\frac{{AC}_TTheo}{{(\left[{Cl}^-\right]}_m\times m_m)+{(\left[{Cl}^-\right]}_w\times V_w)}$$

The theoretical total shoot Cl- accumulation is calculated as follows:

5 $${AC}_TTheo=\sum\nolimits_{ti=0}^{65}({\left[{Cl}^-\right]}_{t65}-{\left[{Cl}^-\right]}_{t-15})\times{mb}_{ti}$$

With:

Rt, the theoretical chloride removal rate in a given microcosm (in %).

$\small {AC}_{T} Theo$, the theoretical total shoot Cl^−^ accumulation (in mg per microcosm) assuming that all the plants have the final Cl^−^ concentration measured at t65,

[Cl^−^]_m_ the initial Cl^−^ concentration in substrate of the microcosm (mg.kg^−1^),

m_m_ the mass of the substrate in the microcosm (kg),

[Cl^−^]_w_ the Cl^−^ concentration in the irrigation water (mg.L^−1^),

V_w_ the volume of irrigation water added into the microcosm throughout the experiment (L).

### Statistical analysis

All the analyses were carried out using R software (R Core Team 2018; RStudio Team 2016) (versions 3.5.1 and 1.1.456 respectively). Significant differences in survival rates were tested following the Neyman and Person approach (*p* = 0.05). Data were analysed with the *survival* package (Therneau and Grambsch 2000; Therneau 2015), and curves were plotted with the *survminer* package (Kassambara and Kosinski 2018). Multiple linear regressions were used to determine the impact of the cultivation duration, concentration and growth stage on the shoot water content and their Cl^−^ concentration. Interactions between parameters and the final minimal adequate models were obtained by backward elimination of non-significant variables (*p* < 0.05). The normality of the residuals was checked prior to interpretation, while correlations between variables were tested with the Pearson’s correlation test; both of these tests were taken from the *stats* package (R Core Team 2018). The *multcompView* package (Graves et al. 2015) was used to show significant distinction at p = 0.05 threshold on graphics developed with the *ggplot2* package (Wickham 2016).

## Results

### Survival rate

**Fig. 2:**
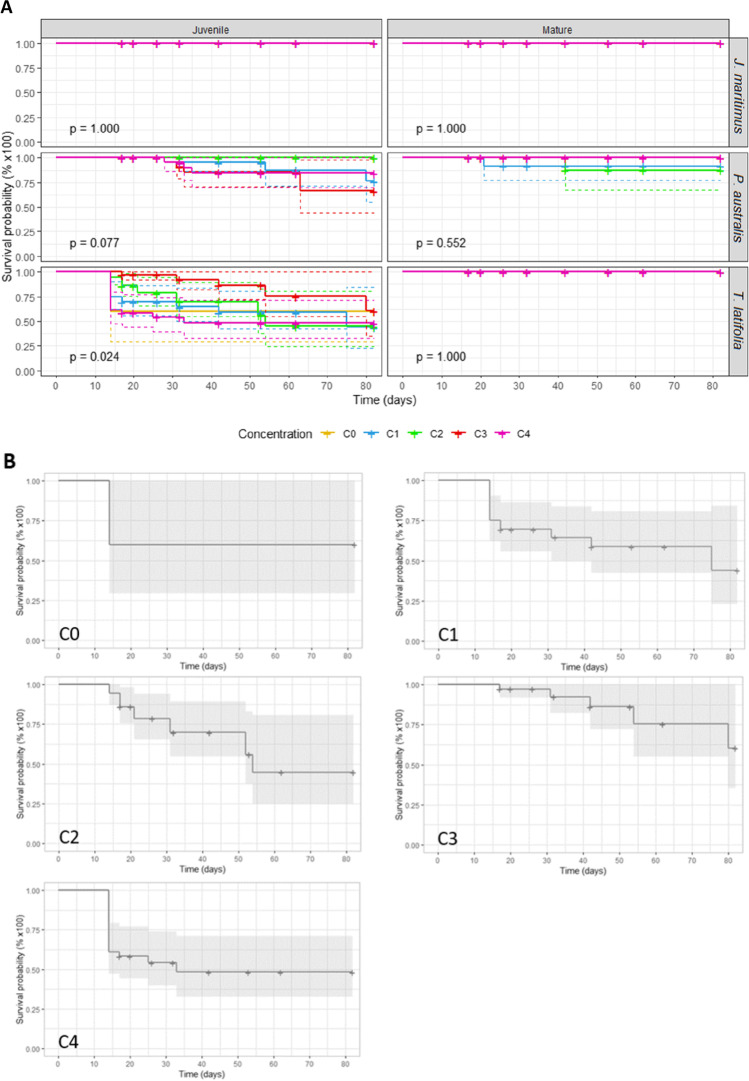
**A**. Survival rates of the three studied species according to their growth stages (mature, m., juvenile, j.) and to the Cl-spiked substrate (in mg.kg^−1^ Cl^−^ and ‰ NaCl: C0 = 0; C1 = 1,875 mg.kg^−1^, 3‰; C2 = 3,750 mg.kg^−1^, 6‰; C3 = 7,500 mg.kg^−1^, 12‰; C4 = 15,000 mg.kg^−1^, 24‰). **B**. Zoom on the survival rates of juvenile specimens of *T. latifolia* with detailed graphic at each tested concentration. Rates are calculated based on the Kaplan Meier estimator. Confidence intervals are represented in dotted lines, cross marks correspond to censured data (sampled plants)

Plant survival was monitored throughout the experiment. All the mature plants of the three macrophytes yied survival rates ranging between 88 and 100% (Fig. 2; Supplementary Table 2). The mature plants of *J*. *maritimus* and *T. latifolia* show excellent survival rates of 100% whatever the salinity (from C0 to C4) whereas *P. australis* yield different results according to microcosms, with values of 100% for C0, C3 and C4, 92 ± 8% for C1 and 88 ± 12% for C2 (Fig. 2). Under similar conditions, juvenile plants show slightly higher mortality for *P. australis* and *T. latifolia*, but have the same survival rate (i.e., 100%) as the mature plants of *J. maritimus* whatever the salinity. The juvenile *T. latifolia* plants display the lowest survival rates, with values of 60 ± 22% for C0, 44 ± 15% for C1, 45 ± 13% for C2, 60 ± 17% for C3 and 48 ± 10% for C4. Intermediate survival rates are found for the juvenile plants of *P. australis*, i.e.100% in both C0 and C2 microcosms and slightly lower in C1, C3 and C4, with values of 76 ± 13%, 66 ± 13 ± and 85 ± 8% respectively.

### Shoot water content

**Fig. 3:**
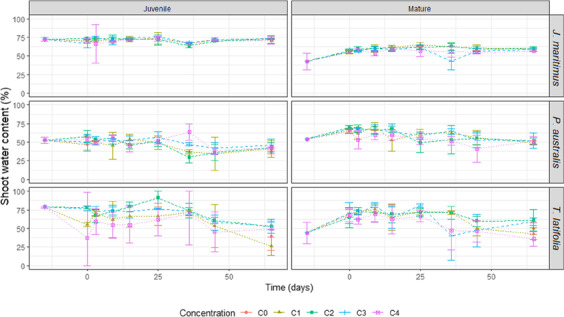
Shoot water contents (%) of the three studied species according to the growth stages (mature, m., juvenile, j.) in the different Cl-spiked microcosms (in mg.kg^−1^ Cl^−^ and ‰ NaCl: C0 = 0; C1 = 1,875 mg.kg^−1^, 3‰; C2 = 3,750 mg.kg^−1^, 6‰; C3 = 7,500 mg.kg^−1^, 12‰; C4 = 15,000 mg.kg^−1^, 24‰). Error bars represent variability among replicates

Shoot water contents were evaluated at day 3, 9, 15, 25, 36, 45 and 65*.* Similar values of shoot water contents in *J. maritimus* are measured whatever the cultivation duration and substrate chloride content for each growth stage considered separately (Fig. 3) (Table 1). However, significant differences are observed according to the plant growth stage: the average water content of the juvenile plants of *J. maritimus* is higher, ca. 71.3 ± 5.1% (2σ), compared with the mature plants, ca. 56.3 ± 13.0% (2σ). For *P. australis*, shoot water content also varies depending on the growth stage, with 48.1 ± 14.7% (2σ) at the juvenile stage and 58.2 ± 14.6% (2σ) at the mature stage. For each salinity modality, a slight decrease of the shoot water content is observed over the experiment duration. However, due to measurements uncertainties this decrease cannot be considered as significant. Moreover, values are similar to those measured in the control microcosms (C0) at t0 and at the end of the experiment (t65). When considering water contents of the *T. latifolia* shoots, high variations are observed over time, with dependence on growth stages in all Cl^−^-spiked microcosms. Even at a same sampling time, a large variability is observed between the triplicate measurements. All microcosms combined, shoot water contents range from 26 ± 12% to 91 ± 14% without any clear tendency either among growth stages or among chloride spiked media (respective variances of 191 and 163 for juvenile and mature stages) (Fig. 3). In the control C0 microcosms, a constant water content is measured in shoots of mature *T. latifolia* over the experiment duration (44 ± 14% at t0; 50 ± 8% at t65) whereas a clear decrease is observed in the juvenile specimens (78 ± 1% at t0, 40 ± 19% at t65).

**Table 1:** Average shoot water contents (%) of the studied plants with standard deviation (1σ) (*n* = 3 for each value). *NS* not sampled

Time (j)	water % *J. maritimus*									
	Juvenile					Mature				
	C0	C1	C2	C3	C4	C0	C1	C2	C3	C4
-15	73 ± 2					42±11				
0	NS	70 ± 4	73 ± 1	67 ± 6		NS	55 ± 3	56 ± 3	57 ± 1	56 ± 2
3	NS	70 ± 1	73 ± 0	74 ± 2	67 ± 26	NS	59 ± 4	59 ± 3	57 ± 1	58 ± 1
9	NS	72 ± 3	73 ± 5	70 ± 5	69 ± 1	NS	60 ± 5	60 ± 4	60 ± 2	53 ± 3
15	NS	73 ± 4	72 ± 1	74 ± 1	72 ± 2	NS	62 ± 1	61 ± 4	59 ± 3	60 ± 2
25	NS	73 ± 9	72 ± 6	76 ± 1	73 ± 3	NS	65 ± 3	62 ± 2	62 ± 3	56 ± 7
36	NS	68 ± 2	63 ± 3	68 ± 1	67 ± 2	NS	62 ± 4	63 ± 5	42 ± 11	56 ± 7
45	NS	72 ± 2	70 ± 1	72 ± 2	72 ± 1	NS	61 ± 4	59 ± 4	57 ± 3	55 ± 3
65	73 ± 0	73 ± 1	72 ± 5	73 ± 1	71 ± 5	59 ± 2	59 ± 2	61 ± 2	58 ± 2	56 ± 1
Time (j)	water % *P. australis*									
	Juvenile					Mature				
	C0	C1	C2	C3	C4	C0	C1	C2	C3	C4
-15	53 ± 4					54 ± NA				
0	NS	47 ± 9	58 ± 8	50 ± 11	53 ± 5	NS	66 ± 3	c	68 ± 0	64 ± 3
3	NS	50 ± 2	54 ± 3	52 ± 5	49 ± 3	NS	62 ± 8	68 ± 5	67 ± 2	53 ± 12
9	NS	45 ± 18	55 ± 4	51 ± 4	55 ± 5	NS	71 ± 6	66 ± 5	68 ± 6	60 ± 6
15	NS	54 ± 7	47 ± 5	52 ± 6	44 ± 8	NS	53 ± 15	67 ± 4	64 ± 11	59 ± 7
25	NS	49 ± 9	51 ± 6	57 ± 7	52 ± 5	NS	62 ± 5	49 ± 13	59 ± 5	55 ± 9
36	NS	37 ± 3	29 ± 8	47 ± 2	63 ± 11	NS	63 ± 5	53 ± 19	65 ± 4	52 ± 6
45	NS	35 ± 22	36 ± 11	42 ± 7	38 ± 5	NS	56 ± 10	55 ± 9	53 ± 10	41 ± 18
65	37 ± 3	41 ± 11	43 ± 6	46 ± 9	43 ± 1	49 ± 2	49 ± NA	52 ± 4	52 ± 10	52 ± 5
Time (j)	water % *T. latifolia*									
	Juvenile					Mature				
	C0	C1	C2	C3	C4	C0	C1	C2	C3	C4
-15	78 ± 1					44 ± 14				
0	NS	55 ± 3	78 ± 2	76 ± 3	37 ± 61	NS	70 ± 6	64 ± 13	70 ± 14	68 ± 9
3	NS	71 ± 4	67 ± 9	73 ± 6	59 ± 17	NS	73 ± 2	73 ± 5	68 ± 6	62 ± 6
9	NS	62 ± 24	73 ± 8	73 ± 5	55 ± 18	NS	75 ± 3	71 ± 12	81 ± 3	70 ± 12
15	NS	66 ± 15	79 ± 6	73 ± 9	55 ± 24	NS	65 ± 17	69 ± 11	64 ± 14	62 ± 20
25	NS	67 ± 12	91 ± 14	76 ± 1	62 ± 22	NS	73 ± 5	71 ± 5	80 ± 2	66 ± 8
36	NS	71 ± 4	74 ± 10	73 ± 5	69 ± 49	NS	72 ± 2	71 ± 9	40 ± 33	47 ± 26
45	NS	53 ± 29	61 ± 4	58 ± 10	45 ± 27	NS	50 ± 10	60 ± 7	47 ± 22	46 ± 14
65	40 ± 19	26 ± 12	53 ± 9	53 ± 5	49 ± 11	50 ± 8	43 ± 7	61 ± 15	59 ± 5	36 ± 10

### Shoot chloride contents

**Fig. 4:**
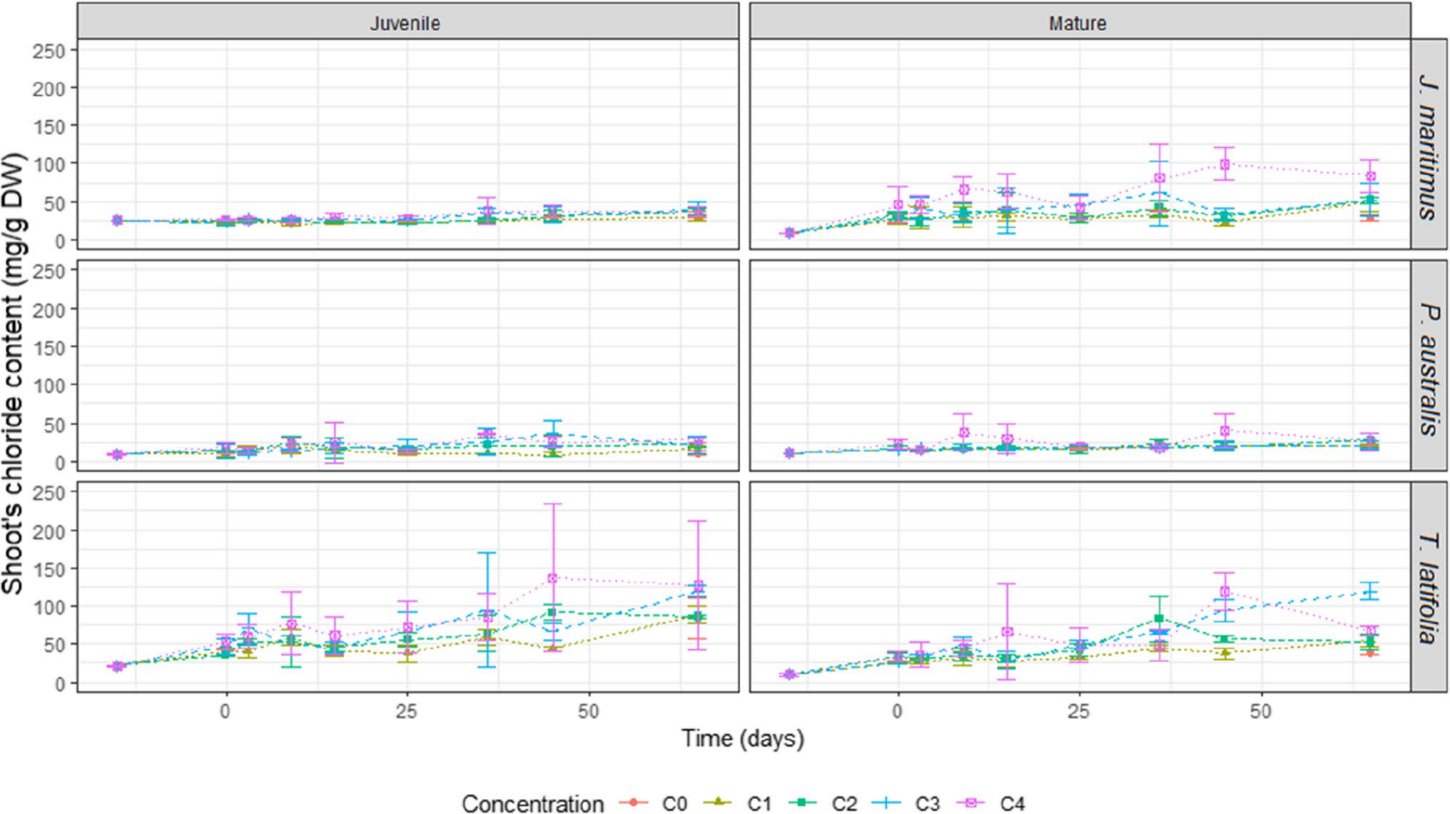
Shoot chloride contents (mg.g^−1^ DW) according to the growth stages (mature, m., juvenile, j.) in the different Cl-spiked microcosms (in mg.kg^−1^ Cl^−^ and ‰ NaCl: C0 = 0; C1 = 1,875 mg.kg^−1^, 3‰; C2 = 3,750 mg.kg^−1^, 6‰; C3 = 7,500 mg.kg^−1^, 12‰; C4 = 15,000 mg.kg^−1^, 24‰). Error bars represent variability among replicates

Shoot chloride contents were evaluated at each sampling date. At the start of the experiment, the shoot Cl^−^ contents of plants are low in the mature specimens of all three macrophytes (9–10 mg.g^−1^ DW, mean values on triplicates), but can reach higher values in specimens considered at their juvenile stage, i.e., 20–25 mg.g^−1^ DW. Only juvenile *P. australis* specimens show low Cl^−^ contents identical to the values found in mature plants. Chloride contents are inversely correlated with the plant biomass being lower in juvenile microcoms, except for *P. australis* which shows no significant variation in biomass between juvenile and mature specimens (Table 2 and supp data). Considering the evolution of Cl^−^ contents over the experiment duration, two groups of plants can be distinguished: (i) plants whose shoots chloride contents remain stable or only increase slighty with time, values at t65 being 1 to at the most 3 times higher than those at t0 (i.e. juvenile and mature *P. australis* plants and juvenile *J. maritimus* plants). Maximum Cl^−^ contents of 25–35 mg.g^−1^ are measured in these plants at the end of the experiment. (ii) Plants characterized by shoot chloride contents that increase significantly over the experiment duration; at t65, these contents can reach values 4 to 10 times higher than initial contents (i.e. juvenile and mature *T. latifolia* plants and mature *J. maritimus*). Maximum Cl^−^ contents of 100 to 120 mg.g^−1^ are measured in *T. latifolia*, and of 84 mg.g^−1^ in *J. maritimus*. Mature *J. maritimus* plants show the greatest increase in Cl^−^ shoot contents between t0 and t65 (× 10) (Fig. 4 and Table 2). Chloride shoot contents of macrophytes cultivated in Cl^−^-spiked microcosms are significantly higher than those measured in the control C0 microcosms (Table 2), except for juvenile *J. maritimus* that displays same Cl^−^ accumulation in all microcosms whether saline or non-saline. In shoots of mature *J. maritimus* and juvenile *P. australis*, Cl^−^ contents are significantly higher than the values obtained in the control microcosms (3 times higher). For juvenile and mature *T. latifolia* plants, the values obtained in shoots cultivated in the non-saline microcosms (C0) are surprisingly high and of the same order of magnitude as in spiked microcosms.

**Table 2:** Shoot chloride contents of the aboveground biomass collected at each sampling time. Values are expressed in mg.g^−1^ DW. Three replicates were sampled at each studied time; average values are given for the three replicates

Time (days)	C0	C1	C2	C3	C4
* J. maritimus* Juvenile					
-15	25.1	25.1	25.1	25.1	25.1
0		21.8	24.2	23.2	25.7
3		25.1	25.0	26.9	26.3
9		21.5	25.3	24.7	25.5
15		22.6	23.3	27.5	30.3
25		21.6	22.9	25.2	28.2
36		24.9	25.3	34,0	37.4
45		27.0	31.7	33.1	37.2
65	33.6	28.7	36.1	39.5	37.6
*65: Ci/C0*		0.85	1.1	1.2	1.1
*R* = *t65 / t0*	1.3	1.1	1.4	1.6	1.5
* P. australis* Juvenile					
-15	9.03	9.03	9.03	9.03	9.03
0		10.3	13.6	13.6	17.3
3		14.7	14.3	11.8	14.2
9		20.2	25,0	14.3	21.1
15		13.8	17.4	16.8	24.1
25		10.6	15.7	20.5	13.3
36		9.47	20.7	25.1	35.3
45		8.77	19.6	36.9	25.2
65	10.7	15.2	21.5	20.8	27.6
*65: Ci/C0*		1.4	2.0	1.9	2.6
*R* = *t65 / t0*	*1.2*	1.7	2.4	2.3	3.1
* T. latifolia* Juvenile					
-15	21.1	21.1	21.1	21.1	21.1
0		41.0	35.3	46.6	53.3
3		41.1	52.5	70.4	59.7
9		58.3	52.8	49.2	77,0
15		40.7	46.5	45.6	61.2
25		37.9	55.8	65.1	72.3
36		59.8	63.6	94.7	85.8
45		43.6	91.1	66.9	137
65	83.8	88.1	85.6	84.7	127
*65: Ci/C0*		1.1	1.0	1.0	1.5
*R* = *t65 / t0*	*4.0*	4.2	4.1	4.0	6.0
* J. maritimus* Mature					
-15	8.6	8.6	8.6	8.6	8.6
0		26.6	32.1	29.4	46.2
3		28.6	23.6	40.4	44.3
9		29.5	35.3	31.3	65.5
15		31.1	38.3	38.1	63.3
25		26.8	29.6	44.3	42.1
36		33.2	41.5	61.2	80.9
45		22.6	30.6	32.8	98.7
65	28.7	49.1	51.9	51.9	83.6
*65: Ci/C0*		*1.7*	*1.8*	*1.8*	*2.9*
*R* = *t65 / t0*	*3.4*	*5.7*	*6.1*	*6.1*	*9.8*
* P. australis* Mature					
-15	10.7	10.7	10.7	10.7	10.7
0		15.2	16.8	15.5	22.5
3		13.6	14.7	15.9	14.3
9		16.7	15.4	18.3	37.3
15		16.0	17.5	16.4	29.9
25		16,0	15.2	17.3	19.0
36		17.2	21.6	17.6	18.3
45		21,0	18.8	21.0	39.9
65	18.4	19.1	27.6	21.2	25.5
*65: Ci/C0*		1.0	1.5	1.1	1.4
*R* = *t65 / t0*	1.7	1.8	2.6	2.0	2.4
* T. latifolia* Mature					
-15	9.4	9.4	9.4	9.4	9.4
0		26.5	33.3	27.0	33.7
3		26.5	29.8	31.6	36,0
9		31.3	36.1	48.9	44.7
15		28.3	31.9	28.7	65.5
25		32.6	42.8	49.3	48.5
36		44.9	83.3	65.1	48.5
45		37.5	56.2	94.1	119
65	39.1	55.5	52.8	119	65.2
*65: Ci/C0*		1.4	1.4	3.0	1.7
*R* = *t65 / t0*	4.1	5.9	5.6	12	6.9

## Discussion

### *Tolerance to Cl*^*−*^* exposure*

Survival rates measured in the studied spiked-microcosms provide information on the salinity tolerance of the three macrophyte species as a function of their growth stage. Firstly, a comparison with the control microcosms allows to discriminate the intrinsic or extrinsic bias of the experiment. For instance all control microscoms show excellent survival rates except those of *T. latifolia* cultivated at its juvenile stage. This highlights the presence of other stresses than salinity that need to be considered in the following discussion. At their mature growth stage, individuals of the three macrophyte species do not show any signs of salinity stress up to 24 ‰ NaCl and 15,000 mg.kg^−1^ Cl^−^ (Fig. 2). This is in agreement with literature on *J. maritimus*, which is described as being tolerant to high salinity; this species is documented to survive at least up to 30 ‰ NaCl (Boscaiu et al. 2011, 2007), the salinity condition of the studied C4 microcosm studied here. However, this is somewhat surprising for *T. latifolia*, which is a species mostly encountered in habitats with moderate salinity and known to be less resistant (Hootsmans and Wiegman 1998; Smith 1986; Jesus et al. 2014). In this study, mature *T. latifolia* plants grow perfectly even at 24 ‰ NaCl. Although *P. australis* is known to be tolerant to a large range of salinity levels even with large variation at a local and at a regional scale due to the effets of climate and substrate clay content (Lissner and Schierup 1997; Lissner et al. 1999a, b; Mauchamp and Mésleard 2001; Burdick et al. 2001; Batriu et al. 2015; McSorley et al. 2016a, 2016b), this study shows that this species displays slightly lower survival rates at its mature stage. However, variations cannot be considered as significant when taking into account statistical parameters (*p* > 0.05). Moreover, the low survival rates are mainly observed in the low salt treatments (C1 and C2), which, according to the literature, are the most suitable conditions for the culture of this species, i.e., around 5 ‰ (Lissner and Schierup 1997; Hartzendorf and Rolletschek 2001)*.* In contrast to mature plants, juvenile specimens of *T. latifolia* and *P. australis* show significantly lower survival rates. While the data cannot be interpreted as resulting from salinity stress for juvenile *T. latifolia*, they highlight a salinity stress for *P. australis* when immature. In the contrary, *J. maritimus* can adapt to Cl^−^ exposure with a 100% survival rate whatever the plant growth stage (Fig. 2). These results may reflect physiological mechanisms that differ between facultative and obligate halophytes.

The water content of a plant is also an indicator of its tolerance to the growing environment (Matoh et al. 1988; Lissner et al. 1999a, b). In combination with the survival data, the water content of the shoots is discussed here in relation to their tolerance to the saline media. Most of the measured values indicate a constant state of the shoot water content in the studied plants whatever the microscosms, salinity level or duration of cultivation, except for *T. latifolia* which shows a significant disturbed distribution as a function of time in a same microcosm (Fig. 3). Once again, the data show that the disturbance of the *T. latifolia* juvenile microcosms cannot be attributed to salinity alone since the C0 control systems behave the same. Except this system, all plants grow optimally in non-saline and in Cl^−^-spiked environments up to 15,000 mg.g^−1^. In the literature, a decrease of the shoot water content of *J. maritimus* and *P. australis* is reported for saline environments (Lissner et al. 1999a; Asaeda et al. 2003; Matoh et al. 1988). It is assumed to be related to an adaptation mechanism, with the purpose of reducing the osmotic potential while maintaining the turgor pressure (Turner and Jones 1980).

To constrain the potential parameters affecting shoot water content, a multiple linear regression was performed using three variables: (i) cultivation duration, (ii) medium salinity, and (iii) plant growth stage (Table 3). This regression also helps analyse the interactions between the variables (Table 3). As a result, it can be observed that the water content of *J. maritimus* is not affected by the Cl^−^ concentration of the growing substrate. Even with a 100% survival rate at its mature growth stage, *P. australis* shows some signs of weakening and is slightly affected by the salinity (slope = 9.10^–4^, *p* < 0.05, and C*G slope = -10.10^–5^, *p* < 0.05) (Table 3). For this species, an adaptation mechanism by water content control can be proposed. For *T. latifolia*, no significant relation exists between the substrate Cl^−^ concentration and its water content (or its survival rate). The behaviour of this species is not exclusively linked to the Cl^−^ exposure as outlined above. The multiple linear regression also highlights a relationship between the shoot water content and plant growth stage for *J. maritimus* and *P. australis* (also observed in Fig. 3). Based on the elapsed time of pre-culture (taking a numerical value of ‘6’ for juvenile -6 months, and ‘12’ for mature -1 year), a slope of -0.8 ± 0.2% / month old (*p* < 0.001) and of 2.4 ± 0.4% / month (*p* < 0.001) can be measured for *J. maritimus* and *P. australis* respectively. The cultivation duration would appear to influence the *P. australis* water content (slope = -0.16 ± 0.03%/day, *p* < 0.001) while it has no effect on the *J. maritimus*. In summary, the multiple linear regression method confirms that the shoot water contents of *J. maritimus* and *P. australis* depend on the age of plant in all treatments, whereas no systematic relation exists regarding the media Cl^−^ concentration. The shoot water contents of *T. latifolia* is not linked with the three variables studied here: Cl^−^ exposure level, plant age, and cultivation duration. The behaviour of this species needs to be explained by at least some parameter other than salinity.

**Table 3:** Coefficients of a multiple linear regression between the plant water content and the cultural duration (CD), the growth stage (G), and the substrate chloride salinity in mg.g-1 (C) and their interactions. *ns* non-significant, * = *p* < 0.05, ** = *p* < 0.01, and *** = *p* < 0.001. No-significant terms were withdrawn from the multiple linear regression calculation

Shoot water contents			
Variables	*J. maritimus*	*T. latifolia*	*P. australis*
Intercept	67.2 ± 1.3 ***	80.8 ± 5.7 ***	34.9 ± 3.7 ***
Culture duration (CD) % / day	ns	-0.6 ± 0.2 **	-2.10^–1^ ± 3. 10^–2^ ***
Cl salinity (C) % / mg.kg^−1^	ns	ns	9.10^–4^ ± 4. 10^–4^ *
Growth stage (G) Mathematical approach % / month	-0.8 ± 0.2 ***	-1.5 ± 0.6 *	2.4 ± 0.4 ***
Interactions			
CD*G % / day. month old	8.10^–3^ ± 4.10^–3^ *	4.10^–2^ ± 2.10^–2^ *	ns
CD*C % / day. mg.kg^−1^	ns	ns	ns
C*G % / mg.kg^−1^. month old	ns	ns	-10.10^–5^ ± 5.10^–5^ *
CD*C*G % / day. mg.kg^−1^. month old	ns	ns	ns

### Shoots chloride accumulation

For the three macrophytes, a slight increase of the shoot Cl^−^ concentration is observed in the control microcosms (C0) from t0 to t65 (*t* test, *p* < 0.001). This increase is assigned to the Cl^−^ supplied to the microcosms due to irrigation with water containing 62.7 ± 0.5 mg.L^−1^ Cl^−^ (Supplementary Table 3).

At day 65, only mature specimens of *J. maritimus* and juvenile individuals of *P. australis* have shoot Cl^−^ contents significantly higher in Cl^−^-spiked microcosms than in the controls (C0). This highlights a mechanism of uptake, translocation and sequestration of the Cl^−^ ions in shoots that is specific to both species for the considered growth stage. By contrast, neither *J. maritimus* at its juvenile stage nor *P. australis* at its mature stage demonstrate a high Cl^−^ sequestration potential, since their shoot Cl^−^ contents are close to those measured in the control microcosm. The behaviour of these plants is governed by a mechanism of chloride exclusion by roots, pointing out the importance of variations in plant metabolism as a function of plant growth stage. Although it would be interesting to investigate the metabolic mechanisms in more detail, the purpose of this study remains focused on evaluating the impact of these phenomena on the desalinization potential of the selected species. *T. latifolia* shows a high capacity of Cl^−^ sequestration that is surprisingly also demonstrated in non-saline media (control C0 microcosms). It is as if the plant is able to take up chloride ions, not only those added to the system but also those intimately linked to the mineral substrate. *P. australis* is a Cl^−^ accumulator species, with juvenile plants being able to accumulate chloride in their aerials parts up to a maximum critical threshold value of around 25 mg.g^−1^ DW. This is in agreement with the values of 24 mg.g^−1^ DW reported in literature (McSorley et al. 2016b). *J. maritimus* is also documented as exhibiting shoot chloride accumulation at levels of ca. 20 mg.g^−1^ DW (Al Hassan et al. 2016); such levels are indeed measured in our experiments with juvenile plants. However, mature specimens of *J. maritimus* are found to be better accumulators than juveniles, with shoots Cl^−^ contents of around 85–90 mg.g^−1^ DW. Compared to the two other macrophytes, both juvenile and mature *T. latifolia* plants show higher chloride accumulation than expected from literature, with reported values of 24–63 mg.g^−1^ Cl^−^ DW (Morteau et al. 2009; Rozema et al. 2014). To our knowledge, the present study is the first report of such high values of Cl^−^ in *T. latifolia* shoots, reaching levels of ca. 100–120 mg.g^−1^ DW.

### Chloride removal and phytoremediation capacity

**Fig. 5:**
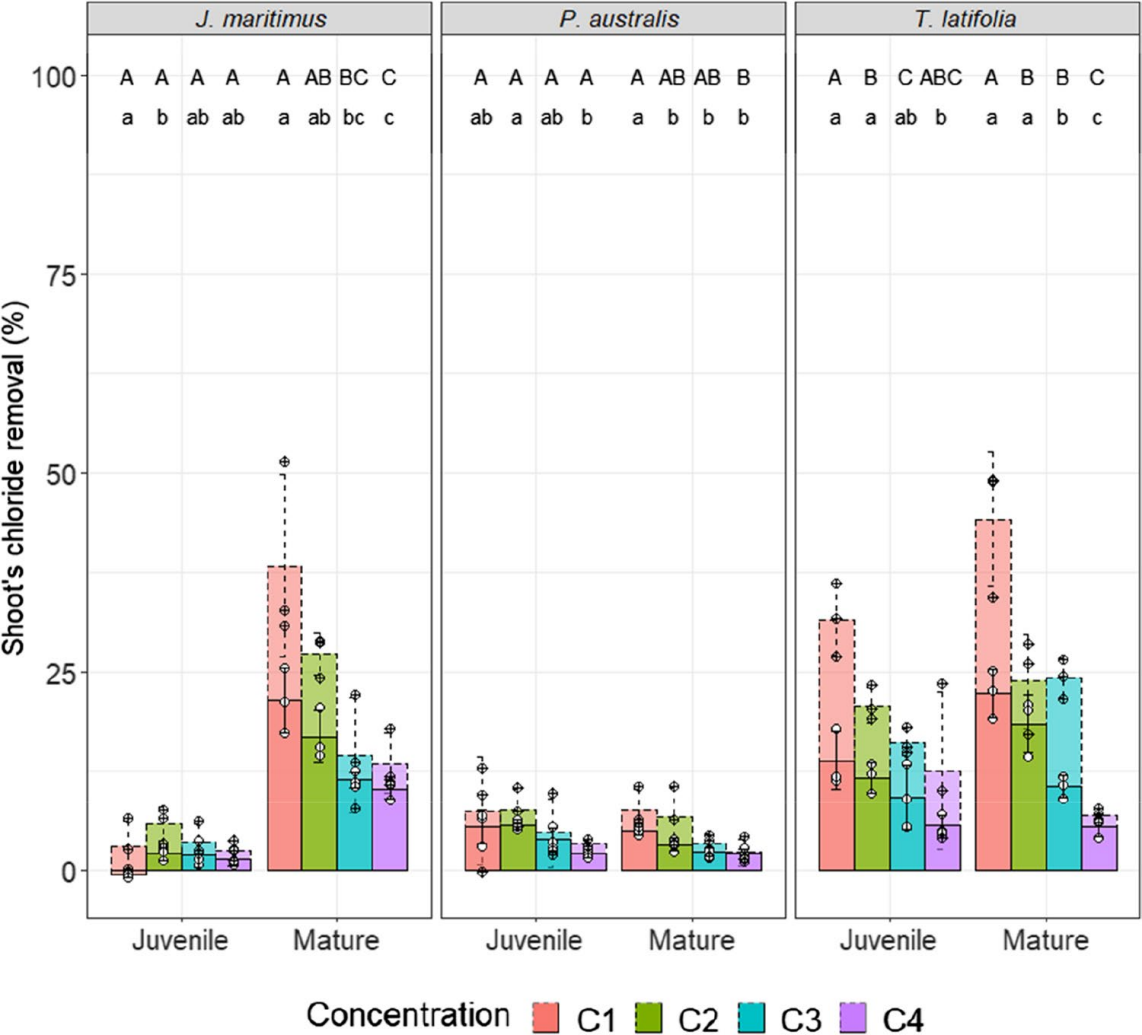
Chloride removal rates from substrates spiked from 1,875 mg.kg^−1^ (C1) to 15,000 mg.kg^−1^ in Cl^−^ (C4): capacity of removal in % of the three studied macrophytes tested at different growth stages (bar = average ± SE, *n* = 3, point = individual value). Full bars with white circles correspond to values achieved with sampled plants while shaded dotted bars with cross represent theoretical values calculated in a scenario with no plants sampling over the experiment duration. C1 = 3‰ NaCl; C2 = 6‰ NaCl; C3 = 12‰ NaCl; C4 = 24‰ NaCl. Letters are used to show significant difference between mean value with Student t-test at a threshold of *p* = 0.05

The phytoremediation potential of the studied plants and mainly those identified as chloride accumulators can be assessed by taking into account not only shoot Cl^−^ contents in mg.g^−1^ DW but also the effective biomass of the species. By considering the 12 to 9 individuals making up the studied microcosms, Cl^−^ removal rates can be expressed in terms of the proportion (%) of chloride ions in the substrate removed by the harvested shoots, as determined according to Eqs. 3, 4, and 5 (Fig. 5). In all treatments, the removal rate is inversely correlated with the Cl^−^ amount in the substrate, decreasing when the substrate Cl^−^ contamination increases. However, while the salinity increases by a factor of 2 (from C0 to C4), the removal is only reduced of a few percents. Hence, the greater the Cl^−^ contamination of the substrate, the higher the phytoextraction. The removal rate ranges from a few percent in the *P. australis* microcosms and can reach 20–25% in the mature *J. maritimus* and *T. latifolia* microcosms (Fig. 5). As mentioned previously, neither the mature and juvenile *P. australis* plants, nor the juvenile *J. maritimus*, are able to remediate efficiently the salinity of their substrate whatever the Cl^−^-spiked conditions (rate < 10%). Mature individuals of *J. maritimus* and *T. latifolia* are the most suitable species for a phytodesalinization process. This result is of interest as *J. maritimus* is less often studied in phytoremediation processes as it is known to produce low biomass (less than 1 kg.m^−2^.y^−1^) compared to *P. australis* and *T. latifolia* (between 1 kg.m^−2^.y^−1^ and 5.5 kg.m^−2^.y^−1^) (Coon et al. 2000; Ennabili et al. 1998; Ennabili and Ater 2005; Rozema et al. 2016; Guesdon et al. 2016; McSorley et al. 2016b).

However, in the present study, chloride removal rates were determined in microcosms in which plants were sampled over time. As a result, the removal rates are underestimated. They would have been higher if no plants had been removed from the microcosms. To draw up a scenario with no plant sampling over 65 days (closer to a realistic scenario of phytoremediation), “theoretical” removal rates were calculated, assuming that all the biomass of a given microcosm have the shoot Cl^−^ contents measured in samples at day 65. Figure 5 reports these theoretical removal rates (%) as a shaded doted bar. Compared to those previously discussed, the same trends are observed according to plant species and Cl^−^ exposure. However, the Cl^−^ fraction phytoextracted from the substrate is almost doubled and can reach 50% when considering mature *J. maritimus* and *T. latifolia*. Net Cl^−^ removal rates would be substantially higher by assuming that a full-scale phytoremediation process would be dimensioned with plants cultivated over a duration of several months to a year with a harvest once a year. In this way, we can demonstrate the effectiveness of the phytoremediation of the Cl^−^-spiked substrate for mature *J. maritimus* and *T. latifolia* plants. Cl^−^ accumulation in roots was not investigated in this study. However, *P. communis* is known to have high root Cl^−^ concentrations (Matoh et al. 1988) suggesting that the substrate remediation could be also achieved by combining the shoot Cl^−^ removal (this study) with Cl^−^ sequestration in roots. Moreover, root growth is less disturbed by the salinity than is the case for the aerial plant parts (Adam 1990; Cheeseman 1988). It would be interesting to determine the Cl^−^ amounts accumulated in the roots of the studied macrophytes to assess their global capacity to remediate Cl^−^ enriched media.

## Conclusion

Oldest (mature) plants of all three selected species are shown to have the higher survival rates, underlying the need to take into account the growth stage of plants when dealing with Cl^−^ phytoremediation. *P. australis* shows a specific range of salinity resistance, being well adapted to medium saline environments with a low chloride phytoextraction potential at both growth stage. By contrast, the potential for chloride extraction and translocation into aerial parts of *J. maritimus* and *T. latifolia* is demonstrated. For *J. maritimus*, this innovative result implies that the efficiency of the phytoextraction mechanism varies as a function of the plant growth stage with the oldest specimens having higher accumulation and removal capacities. Taken together, the combined data lead us to a realistic evaluation of chloride removal and indicate that *J. maritimus* followed by *T. latifolia* seem to be the best adapted species to remediate chloride salinity from soils or wastes.

## Supplementary Information

Below is the link to the electronic supplementary material.

Supplementary file1 (DOCX 165 KB)

## Data Availability

All data generated or analyzed during this study are included in the published article and supplementary information files. More information is available from the corresponding author on reasonable request.
