# Supplementary material for: Chloride accumulation in aboveground biomass of three macrophytes (Phragmites australis, Juncus maritimus, and Typha latifolia) depending on their growth stages and salinity exposure: application for Cl− removal and phytodesalinization
Source: Environ Sci Pollut Res Int. 2022 Jan 20;29(23):35284–99. doi: 10.1007/s11356-021-17591-3 (PMC9076746; doi:10.1007/s11356-021-17591-3)
Supplement: Supplementary file 1 — Supplementary file1 (DOCX 165 KB) [file 11356_2021_17591_MOESM1_ESM.docx]

***Supplementary data***

**Reference ESPR-D-20-15168**

**Chloride accumulation in aboveground biomass of three macrophytes (*Phragmites australis*, *Juncus maritimus,* and *Typha latifolia*) depending on their growth stages and salinity exposure. Application for Cl^-^ removal and phytodesalinization.**

Emmanuel Delattre, Isabelle Techer, Benjamin Reneaud, Patrick Verdoux, Isabelle Laffont-Schwob, Philippe Prohin

**Corresponding author** : Isabelle TECHER: [isabelle.techer@unimes.fr](mailto:isabelle.techer@unimes.fr)

Supplementary Table 1: Biomass of J. maritimus, P. australis and T. latifolia specimens sampled at each studied time. Values are expressed in grams (g DW)

Supplementary Table 2: Number of dead plants and survival rates determined over the experiment for P. australis and T. latifolia when cultivated either at their juvenile (a) or mature stage (b). J. maritimus was extracted from this table because of no death during the time of experiment. (SR = survival rate, SE = standard error, LCI 95% = lower 95 % confidence interval boundary, UCI 95 % = upper 95 % confidence interval boundary). The parameter plant at risk corresponds to plant still alive at the moment and not censored (sampled).

a-

| ***P. australis*** | | | | | | | ***T. latifolia*** | | | | | | |
| --- | --- | --- | --- | --- | --- | --- | --- | --- | --- | --- | --- | --- | --- |
| ***Juvenile*** | | | | | | | ***Juvenile*** | | | | | | |
| concentration=C0 | | | | | | | concentration=C0 | | | | | | |
|  | | | | | | | Time | Plant at risk | Deaths | SR (%) | SE (%) | LCI 95% | UCI 95% |
|  |  |  |  |  |  |  | 14 | 5 | 2 | 60 | 21.9 | 29.3 | 100 |
| concentration=C1 | | | | | | | concentration=C1 | | | | | | |
| Time | Plant at risk | Deaths | SR (%) | SE (%) | LCI 95% | UCI 95% | Time | Plant at risk | Deaths | SR (%) | SE (%) | LCI 95% | UCI 95% |
| 28 | 21 | 1 | 95.2 | 4.65 | 86.6 | 100 | 14 | 36 | 9 | 75 | 7.22 | 62.1 | 90.6 |
| 54 | 12 | 1 | 87.3 | 8.71 | 71.8 | 100 | 17 | 27 | 2 | 69.4 | 7.68 | 55.9 | 86.2 |
| 80 | 8 | 1 | 76.4 | 12.74 | 55.1 | 100 | 31 | 14 | 1 | 64.5 | 8.58 | 49.7 | 83.7 |
|  | | | | | | | 42 | 11 | 1 | 58.6 | 9.6 | 42.5 | 80.8 |
|  |  |  |  |  |  |  | 75 | 4 | 1 | 44 | 14.59 | 22.9 | 84.3 |
| concentration=C2 | | | | | | | concentration=C2 | | | | | | |
|  | | | | | | | Time | Plant at risk | Deaths | SR (%) | SE (%) | LCI 95% | UCI 95% |
|  |  |  |  |  |  |  | 14 | 36 | 2 | 94.4 | 3.82 | 87.3 | 100 |
|  |  |  |  |  |  |  | 17 | 34 | 3 | 86.1 | 5.76 | 75.5 | 98.2 |
|  |  |  |  |  |  |  | 21 | 23 | 2 | 78.6 | 7.3 | 65.5 | 94.3 |
|  |  |  |  |  |  |  | 31 | 18 | 2 | 69.9 | 8.72 | 54.7 | 89.2 |
|  |  |  |  |  |  |  | 52 | 10 | 2 | 55.9 | 11.26 | 37.7 | 83 |
|  |  |  |  |  |  |  | 54 | 5 | 1 | 44.7 | 13.46 | 24.8 | 80.7 |
| concentration=C3 | | | | | | | concentration=C3 | | | | | | |
| Time | Plant at risk | Deaths | SR (%) | SE (%) | LCI 95% | UCI 95% | Time | Plant at risk | Deaths | SR (%) | SE (%) | LCI 95% | UCI 95% |
| 28 | 21 | 1 | 95.2 | 4.65 | 86.6 | 100 | 17 | 36 | 1 | 97.2 | 2.74 | 92 | 100 |
| 31 | 20 | 1 | 90.5 | 6.41 | 78.8 | 100 | 31 | 20 | 1 | 92.4 | 5.41 | 82.4 | 100 |
| 33 | 17 | 1 | 85.2 | 7.94 | 70.9 | 100 | 42 | 15 | 1 | 86.2 | 7.8 | 72.2 | 100 |
| 63 | 9 | 2 | 66.2 | 13.32 | 44.7 | 98.2 | 54 | 8 | 1 | 75.4 | 12.17 | 55 | 100 |
|  | | | | | | | 80 | 5 | 1 | 60.3 | 16.64 | 35.1 | 100 |
| concentration=C4 | | | | | | | concentration=C4 | | | | | | |
| Time | Plant at risk | Deaths | SR (%) | SE (%) | LCI 95% | UCI 95% | Time | Plant at risk | Deaths | SR (%) | SE (%) | LCI 95% | UCI 95% |
| 28 | 21 | 1 | 95.2 | 4.65 | 86.6 | 100 | 14 | 36 | 14 | 61.1 | 8.12 | 47.1 | 79.3 |
| 33 | 18 | 1 | 89.9 | 6.76 | 77.6 | 100 | 17 | 22 | 1 | 58.3 | 8.22 | 44.3 | 76.9 |
| 35 | 17 | 1 | 84.7 | 8.18 | 70.1 | 100 | 25 | 14 | 1 | 54.2 | 8.62 | 39.7 | 74 |
|  | | | | | | | 33 | 9 | 1 | 48.1 | 9.54 | 32.7 | 71 |

b-

| ***P. australis*** | | | | | | | ***T. latifolia*** |
| --- | --- | --- | --- | --- | --- | --- | --- |
| ***Mature*** | | | | | | | ***Mature*** |
| concentration=C0 | | | | | | | concentration=C0 |
|  |  |  |  |  |  |  |  |
| concentration=C1 | | | | | | | concentration=C1 |
| Time | Plant at risk | Deaths | SR (%) | SE (%) | LCI 95% | UCI 95% |  |
| 21 | 12 | 1 | 91.67 | 7.98 | 77.29 | 100 |  |
| concentration=C2 | | | | | | | concentration=C2 |
| Time | Plant at risk | Deaths | SR (%) | SE (%) | LCI 95% | UCI 95% |  |
| 42 | 8 | 1 | 87.5 | 11.7 | 67.3 | 100 |  |
| concentration=C3 | | | | | | | concentration=C3 |
|  |  | | | | |  |  |
| concentration=C4 | | | | | | | concentration=C4 |
|  |  |  |  |  |  |  |  |

Supplementary Table 3: Total amount of chloride ions in the different studied microcosms considering the initial NaCl addition and the total volume of irrigation. Values are expressed in grams (g).

| Concentration | *Juncus* *maritimus* | | | *Phragmites* *australis* | | *Typha* *latifolia* | |
| --- | --- | --- | --- | --- | --- | --- | --- |
|  | Baby | Juvenile | Mature | Juvenile | Mature | Juvenile | Mature |
| C0 | 0.73 | 0.61 | 1.10 | 0.79 | 1.49 | 0.66 | 1.47 |
| C1 | 1.33 | 3.52 | 5.63 | 4.12 | 5.31 | 4.14 | 5.16 |
| C2 | 1.80 | 5.65 | 9.58 | 6.21 | 9.28 | 6.37 | 8.97 |
| C3 | 2.96 | 10.0 | 16.4 | 10.0 | 16.4 | 10.4 | 16.5 |
| C4 | 4.98 | 19.2 | 32.3 | 19.0 | 31.6 | 19.2 | 33.6 |
